# Assessing the impact of public education on a preventable zoonotic disease: rabies

**DOI:** 10.1017/S0950268817002850

**Published:** 2017-12-22

**Authors:** E. HASANOV, S. ZEYNALOVA, M. GELEISHVILI, E. MAES, E. TONGREN, E. MARSHALL, A. BANYARD, L. M. MCELHINNEY, A. M. WHATMORE, A. R. FOOKS, D. L. HORTON

**Affiliations:** 1 State Veterinary Control Service, Veterinary Department, Baku, Azerbaijan; 2 Republican Veterinary Laboratory, Baku, Azerbaijan; 3 Centers for Disease Control and Prevention South Caucasus Field Epidemiology Training Program, Tbilisi, Georgia; 4 Paul G. Allen School for Global Animal Health, Washington State University, Washington, USA; 5 Animal and Plant Health Agency (APHA), Wildlife Zoonoses and Vector-borne Diseases Research Group, Addlestone, Surrey, UK; 6 Department of Clinical Infection, Microbiology and Immunology, University of Liverpool, Liverpool, UK; 7 School of Veterinary Medicine, University of Surrey, Surrey, UK

**Keywords:** Cluster method, epidemiology, KAP survey, public awareness, rabies, vaccination, zoonosis

## Abstract

Effective methods to increase awareness of preventable infectious diseases are key components of successful control programmes. Rabies is an example of a disease with significant impact, where public awareness is variable. A recent awareness campaign in a rabies endemic region of Azerbaijan provided a unique opportunity to assess the efficacy of such campaigns. A cluster cross-sectional survey concerning rabies was undertaken following the awareness campaign in 600 households in 38 randomly selected towns, in districts covered by the campaign and matched control regions. This survey demonstrated that the relatively simple awareness campaign was effective at improving knowledge of rabies symptoms and vaccination schedules. Crucially, those in the awareness campaign group were also 1·4 times more likely to report that they had vaccinated their pets, an essential component of human rabies prevention. In addition, low knowledge of appropriate post-exposure treatment and animal sources of rabies provide information useful for future public awareness campaigns in the region and other similar areas.

## INTRODUCTION

Preventable communicable diseases remain a major threat for a significant proportion of the global population [1]. Although global average life expectancy is increasing, and the relative impact of infectious diseases is reducing [2, 3], their impact varies with socio-economic factors. These global summary data therefore hide large differences between countries and threaten to prematurely direct resources away from preventable disease control [4].

Rabies is one example of a preventable neglected infectious disease with global distribution and impact [5]. Rabies is an acute viral infectious disease affecting animals and humans, and causes social and public health problems worldwide. In addition to the uniquely high mortality and societal impacts, the disease has a significant global economic burden estimated at $8·6 billion per year [6]. Concerted international control efforts are underway, with significant progress already made in many regions, including North and Latin America, Europe and parts of Asia. The World Health Organization (WHO)/FAO/OIE vision for the elimination of dog-mediated rabies is 2030 [7], thereby contributing to poverty alleviation and a reduction in childhood mortality as part of the Millennium Development Goals [8]. For some regions of the world, the 2030 target remains an ambitious challenge, although many of these regions are constantly striving to expand their ability to combat rabies. The Caucasus region, on the borders of Europe has ongoing rabies complicated by its geographic position and multiple host species [9, 10].

Significant progress has recently been made in the surveillance and reporting of rabies in the Caucasus region, which is an important step towards future control [9, 11]. In Azerbaijan, human rabies is notifiable by law, and both scanning and active surveillance are undertaken for animal rabies. In a recent analysis of surveillance data, the majority of the reported rabies cases were in dogs, although wildlife rabies cases were also reported [12]. Dog rabies vaccination in Azerbaijan is compulsory, but the level of uptake and awareness among owners is unclear, and there are free-roaming dog populations in some areas.

Regional variance in numbers of reported rabies cases throughout Azerbaijan is suggestive of foci of infection in both animals and humans. For example, from 139 registered animal rabies cases in Azerbaijan between 2009 and 2013, 61 (43%) were in one region (Sheki-Belokan) and within that region 48 cases (79%) were registered in just four districts (Sheki, Gakh, Gabala and Oguz) ([9] and unpublished observations). In addition, 42 human rabies cases were reported from the region over the same time period. Due to this high number of cases, in 2013 an awareness campaign was conducted in two districts (Sheki and Gakh) to educate residents on rabies symptoms and prevention.

Public education has an extremely important role in rabies control: including encouraging responsible pet ownership, and ensuring appropriate health service-seeking behaviour following potential exposure [11, 13, 14]. Multiple surveys of public knowledge of rabies have demonstrated better awareness in educated individuals, and in those with direct or indirect experience of rabies exposure [13, 15–20]. The successful campaigns of dog rabies reduction in Latin America have also included important educational components [21–24]. Assessing the efficacy of those educational components can, however, be challenging but is important for prioritising resources in rabies control programmes [25].

To evaluate the campaign effectiveness in Azerbaijan and inform similar studies in other regions, a survey of the population's knowledge, attitudes and practices relating to rabies (KAP) study was conducted in the region following the education campaign. This study provided a unique opportunity to compare the knowledge in the region, to that of populations in neighbouring, demographically similar regions where no awareness campaign had been undertaken, to assess the efficacy of such awareness campaigns and provide evidence for rabies control policy.

## METHODS

### Enhancing public awareness

In 2013, an awareness campaign was conducted in two districts (Sheki and Gakh) to educate residents on rabies symptoms and prevention, to improve surveillance and reduce human cases. The information materials consisted of posters, leaflets and text messages. The posters were designed to include simplified graphical illustrations of key concepts in three separate posters: (i) animal species able to transmit rabies, (ii) signs and symptoms of rabies and (iii) appropriate action in the event of a bite (available on request). Two hundred copies of each poster were distributed to schools, community centres and medical clinics in 50 villages across the districts. An information leaflet was also developed for public and animal health professionals and distributed in Sheki and Gakh through local offices of the Anti-Plague Service. For the telecommunications campaign, a short text message was prepared conveying messages regarding action in the event of a bite and was sent to 800 randomly selected phone numbers in the regions by short messaging service.

### Evaluating the communications strategy

**Fig. 1.**
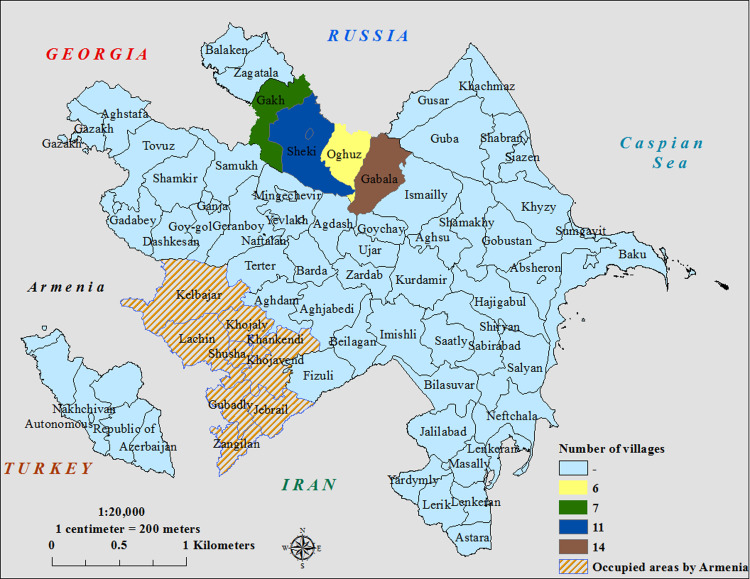
Map showing the locations of the study regions in Azerbaijan, and number of villages surveyed during the study.

A cluster cross-sectional study was conducted in July 2014. Four districts were chosen; two that had been targeted by the public awareness materials (Sheki and Gakh) and two control districts that had not (Gabala and Oguz). The four districts were comparable in demography and population density (Table 1). A survey questionnaire containing household information on animals, knowledge of animal and human rabies and preventive measures was designed based on previous models, and adapted for regional and cultural relevance (see Supplementary material) [11]. To implement the survey, 10 workgroups were assigned. Sample size was calculated for each district for 95% confidence level at 600 households. Ninety-six clusters of seven households in 38 towns and villages in the four districts were selected using probability proportional to size of the population, within the towns and villages of the four districts. The 38 towns and villages were distributed among the districts as follows: seven villages in Gakh district, 11 villages in Sheki district, 14 villages in Gabala district and six villages in Oguz district (Fig. 1). The starting points for each cluster were then picked randomly using a random number table and a numbered grid overlay of the town or village map. Within each selected grid square, the starting point for house selection was the centre of the square followed by selecting houses in a randomly chosen compass direction until the required number of interviews was completed. One respondent per household over >18 years of age was selected. Data were analysed in EpiInfo™ 7. Prevalence rate ratios (PRR) were calculated from 2 × 2 tables using the Statcalc application in EpiInfo, with *χ*^2^ tests used to assess significance.

**Table 1.** Demographic parameters of the study regions and respondents

	Population and area			Percentage of respondents						
	Total area (km^2^)	Total population	Population density pop/km^2^	Males (%)	Education >elementary level (%)	Relevant vocation (%)	Age 17–24 (%)	Age 25–40 (%)	Age 41–60 (%)	Age >60 (%)
Awareness campaign (Gakh and Sheki)	3920	227 400	58	65·5	27	4·8	4·5	33·0	46·1	16·4
No awareness campaign (Oguz and Gabala)	2770	136 500	49	70·2	20	5·4	6·9	29·5	49·5	14·1

Relevant vocation=human health professional, veterinary health professional, farmer (‘shepherd’).

## RESULTS

### Summary of key findings

The awareness campaign group had better knowledge of rabies symptoms (PRR = 1·3; 95% CI 1·1–1·5) and vaccination schedules (PRR = 1·3; 95% CI 1·1–1·4). Crucially, the awareness campaign group were also 1·4 times more likely to report having vaccinated their dogs (PRR 1·4; 95% Cİ 1·1–1·7). No differences were detected in the low level of health-seeking behaviour following a dog bite between the regions, and no difference was detected between rabies sources identified by the two groups.

### Study participants

Among the 672 study participants, 336 (50%) were from the districts where the awareness campaign had been conducted, 456 (68%) were men and the mean age was 45 years old. Specifically, there were 55 respondents (12%) from Gakh, 166 (36%) from Sheki, 80 (18%) from Oguz and 156 respondents (34%) from Gabala, who were men. There were 30 respondents (14%) from Gakh, 86 (40%) from Sheki, 25 (12%) from Oguz and 75 (35%) from Gabala who were women. Only 117 out of 672 respondents (17%) had more than elementary level education compared with 555 (83%) having elementary level education. Those were 20% of all male respondents, and 13% of all female respondents.

A very low proportion of people had any professional links to human medicine – only 2·7% of all respondents. These, and any other professions having any potential background information on rabies due to their occupation (medical workers, veterinary workers and shepherds), constituted approximately 5% of all respondents, with more in the non-campaign group (5·4% *vs.* 4·8%). Respondents were similar in sex and age group between regions where the awareness campaign was conducted and those where campaigns had not been undertaken (Table 1). A higher percentage of respondents in the campaign group (27%) had secondary or higher education than the control group (20%) but when combined with those individuals with relevant occupations, the difference in those who may have better existing knowledge was not significant (32% compared with 26%, *P* = 0·07).

### Self-reported knowledge compared with actual knowledge

There were no significant differences between men and women in the self-reported awareness of rabies. Eighty-nine per cent of men (398) and 79% of women (168) declared a general knowledge of rabies. Those who promoted themselves as being generally aware of rabies were asked whether cure is possible after the onset of clinical signs in humans. Of 398 male respondents considering themselves to have general knowledge on rabies, 36% (143) suggested cure is possible, and 64% (255) suggested that it was impossible or did not know. Out of the 168 female respondents, 37% (62) suggested that cure was possible, and 63% (106) suggested that survival was impossible or did not know.

Male respondents had better factual knowledge of rabies symptoms when compared with female respondents (PRR 1·4; 95% CI 1·2–1·6) and the majority of respondents (58% (251) of the male respondents and 50% (100) of the female respondents) knew rabies can be prevented in animals by regular vaccination (Table 2).

**Table 2.** Responses of participants regarding rabies prophylaxis in dogs

Response to the question: What should be done in order to protect a domestic animal from rabies?	Frequency (male/female)	Per cent (male/female)	95% confidence limits (male/female)	
One time lifelong vaccination	87/39	20/20	16/14	24/26
Regular vaccination, according to the prescription of the vet	251/100	58/50	53/43	63/57
There are no means of animal protection from rabies	5/1	1·2/0·5	0·4/0·0	2·8/2·8
I do not know	78/57	18/29	15/22	22/35
Other	12/3	2·7/1·5	1·5/0·3	4·9/4·3
Total	433/200	100%		

### Rabies reservoirs and vectors

Most respondents considered wild animals, domestic and free-roaming ownerless dogs as sources of rabies. Notably less than half the respondents considered domestic or ownerless cats as sources of rabies. In addition, a high proportion of respondents (30%) did not consider domestic dogs as a source, and a similar proportion were unsure about free-roaming ownerless dogs as a source of rabies virus.

### Effect of public awareness campaign

Respondents in Gakh and Sheki, where the awareness campaign had been undertaken, were 1·3 times as likely to give correct answers concerning rabies symptoms, including hyper-salivation and changes in animal behaviour, in when compared with Oguz and Gabala districts that had not been targeted with awareness materials. Owner-reported vaccination of dogs was also higher, with 80% of 92 dog owners reporting having vaccinated their dog in Gakh and Sheki districts, compared with only 52% of 142 dog owners in Oguz and Gabala (PRR 1·4, *P* = 0·0015).

Levels of self-reported knowledge in the population of Gakh and Sheki districts compared with those in population of Oguz and Gabala districts were similar: 284 people in Gakh and Sheki districts reported having knowledge of rabies, and 48 did not. In the regions where awareness campaigns had not been undertaken, Oguz and Gabala districts, 282 reported having knowledge of rabies, and 54 reported no knowledge (PRR 1·0; 95% CI 0·9–1·1; *P*-value 0·9). The self-reported knowledge in a subset of the population owning animals was also similar between regions (PRR 0·9; 95% CI 0·9–1·1; *P*-value 0·7).

### Information media

**Fig. 2.**
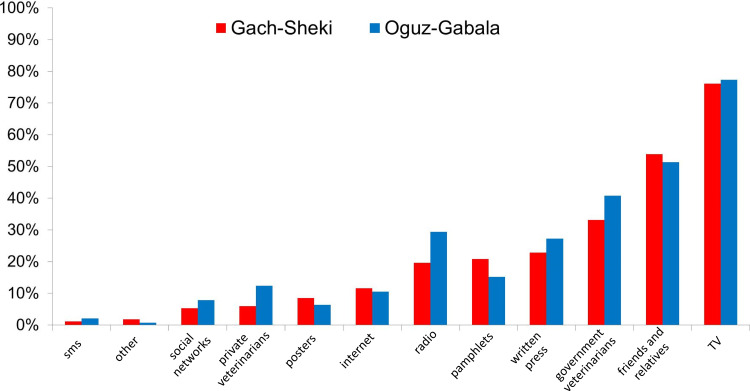
Sources of information used by the respondents. Respondents answer to the question ‘From where do you get information on rabies or other diseases?’ Respondents were able to put more than one answer.

Analysing the informational channels given as the most important sources of information by respondents in Gakh and Sheki districts and Oguz and Gabala districts demonstrated only minor differences. A higher proportion of respondents in the awareness campaign districts mentioned posters and pamphlets, but these differences (under 5%) were dwarfed by the large proportion of respondents who mentioned government veterinarians and other veterinary paraprofessionals (40%), friends (~50%) and television (75%) as the most important sources of information in all regions (Fig. 2).

## DISCUSSION

This rare opportunity to assess the effect of a public education campaign for a neglected disease has demonstrated that the campaign had a detectable positive effect. Vaccination of dogs is a critical component of rabies control, and crucially these data demonstrate that respondents in an area where public awareness was undertaken were more likely to report having vaccinated their dogs. Considering the beneficial effect of increasing vaccination coverage for prevention of disease, this difference is likely to reduce the number of rabies cases in dogs and consequently the number of potential human rabies exposures in the region [26].

In addition, the knowledge increase after the campaign will likely enable an improved recognition of the clinical signs in animals, and improved preventative measures, with the potential for improved post-exposure prophylaxis (PEP) seeking behaviour following exposure. Despite these measurable differences in actual knowledge of rabies, the participants own perception of their knowledge (self-reported awareness) was similar between regions. The majority of respondents in both regions felt that they had good knowledge of rabies. These two findings together raise an important issue, implying that the public may not be aware of their lack of knowledge and may therefore not seek, nor be receptive to, more accurate or up-to-date information [27]. Risk perception, awareness and knowledge are among key determinants of so-called communication inequalities, altering exposure to, and therefore success of public health communication messages [28]. Appropriate health-seeking behaviour is only of benefit if the public have access to health care. In Azerbaijan, PEP is available through the Anti-Plague facilities following WHO guidelines, but this is not the case in all rabies endemic countries.

One explanation for the reported improved dog vaccination is that the awareness campaign served to remind those that already had good knowledge of rabies, to have their dog vaccinated. The concept of priming a population prior to implementing vaccination schemes to improve uptake has a precedent, with successful examples from Africa and Asia [11, 29–31]. A targeted campaign at dog owners may therefore be a more cost-effective method to improve dog vaccination coverage, albeit potentially losing other benefits such as improved health-seeking behaviour and awareness of other rabies source species.

It is possible that historical experience, or unreported cases not captured in recent rabies records influenced respondent knowledge. This was detected in a KAP survey in the Philippines where the greatest effect on knowledge of rabies was associated with having known someone with rabies, albeit in a different epidemiological situation [15]. However, this personal experience might have been expected to influence the level of self-reported knowledge as much or more than the actual factual knowledge of rabies symptoms. In addition, this personal experience was not reflected in the responses of the respondents. There were a slightly higher number of respondents who had secondary or higher education in the awareness campaign group than the control group. Education level has previously been associated with improved awareness of rabies [15, 16, 18]. However, a higher confidence in rabies knowledge (self-reported knowledge) would be expected in this group if education was a major factor. In addition, there were more respondents in the control regions who had veterinary or related occupations.

The KAP survey illustrated a lack of knowledge of rabies reservoirs in the respondents from both groups. These results provide vital information for future campaigns, and illustrate a pressing need to improve knowledge. First, a significant proportion of the population is at risk of not seeking appropriate PEP if bitten by a cat or free-roaming ownerless dog despite both being well recognised as potential sources of rabies [5]. Second, the lack of awareness regarding free-roaming ownerless dogs may affect public engagement in campaigns for responsible dog ownership and control of rabies in free-roaming dogs [32, 33]. A potential limitation of this study is the definition of free-roaming ownerless dogs in the survey. From a rabies prevention perspective, it is relevant whether the animal is owned and/or accessible for vaccination but it was not possible to fully explore the respondents understanding of whether the free-roaming dogs were owned in this study. The perception of a role of wildlife concurs with the reports of rabies cases in foxes [34], but the majority of reported rabies cases are in dogs and domestic animals [9]. This low number of wildlife cases in scanning surveillance is likely due to surveillance bias, and supports more targeted surveillance of wildlife to understand the role of wildlife reservoirs in this epidemiologically complex region [10]. In addition, the historical detection of bat lyssaviruses in the region supports the characterisation of viruses to detect any potential rabies caused by lyssaviruses other than rabies [35, 36].

Although statistically significant and likely to have a positive effect, the effect sizes from the campaign were small, with only a small increase in actual numbers of vaccinated animals and improvement in knowledge. However, given the scope of this education campaign, any detectable positive effect gives confidence that a more comprehensive campaign would have a larger benefit. The sources of information used by the respondents provide extremely useful reference for future campaigns in Azerbaijan, and other socio-economically similar areas. The high proportion (75–80%) of respondents who mentioned television, in contrast to those who mentioned the internet and social media, demonstrates the continued importance of television despite the social media revolution which has influenced other public awareness campaigns [37, 38]. Following television, friends/relatives and government vets were the second and third most frequently cited sources of information. What these data are not able to capture is individuals who see and then read the public awareness materials and then pass on the information to multiple individuals. It is clear that word-of-mouth is an important method of information dissemination; therefore, providing accurate information to targeted respected individuals, such as government vets or community leaders, could have beneficial secondary effects. One very promising approach that was not used here but has proved successful elsewhere is the introduction of rabies awareness in the school curriculum, both establishing a core of knowledge in the young and allowing children to educate the rest of their family [31]. This approach has proven most productive in primary education and where a lecture is combined with printed information [25].

In conclusion, these data provide information with potentially immediate local benefits as well as longer and wider term implications for the rabies situation in Azerbaijan. The public awareness campaign has improved the understanding of key issues relating to rabies exposure and prevention and is therefore suitable for rolling out to other regions in Azerbaijan and with cultural adjustments, to other countries and regions. Improvements highlighted by this study which are being implemented in the ongoing government-led control strategy include optimising the information channels used to distribute the information, and timing campaigns to precede government-led vaccination campaigns. The low level of knowledge regarding animal sources of rabies, and the potential for communication inequalities mean future work could focus on a One Health approach, with the relationships of humans, pets, ownerless dogs and wildlife to better understand the reservoirs and vectors of rabies virus, and further targeting of communication strategies.

## Supplementary material

For supplementary material accompanying this paper visit https://doi.org/10.1017/S0950268817002850. click here to view supplementary material
