# Supplementary material for: Assessing the impact of public education on a preventable zoonotic disease: rabies
Source: Epidemiol Infect. 2017 Dec 22;146(2):227–35. doi: 10.1017/S0950268817002850 (PMC5851038; doi:10.1017/S0950268817002850)
Supplement: Supplementary file 1 [file S0950268817002850sup001.docx]

*Online Supplementary material*

*Questionnaire on study of knowledge of population about rabies (English translation)*

**Demographic Information**

1. **Rayon _______________ (1.1) City /Village ___________________ (1.2)**

**Name, Last name_______________________**

**Telephone_________________**

1. **Sex (2.0)**
2. Male
3. Female
4. **Age _________ (3.0)**
5. **Education (Complete) (4.0)**
6. Elementary
7. High school
8. Vocational
9. Higher (Any University or Graduate School)
10. **Occupation (5.0)**
11. Health care worker
12. Veterinary Doctor
13. Shepherd
14. Other (SPECIFY) ____________________

**Animal Practice**

1. **Do you have any pets or other animals?** (6.0)
2. Yes
3. No (Go to Q8)

| 1. **What species of animals do you have (7.0)** | | **Yes** | **No** | **Unknown** |
| --- | --- | --- | --- | --- |
| **1** | **Do you have any Dogs (7.1)**  ***If “Yes”:* How many dogs? (7.1.1) _______**  ***If “Yes”:* Are they kept in a fenced or walled area? (7.1.2) (Choose one)**   - 1. **Yes, all of them are kept in a fenced or walled area**   2. **Yes, some of them are kept in a fenced or walled area**   3. **No (they can all roam freely)** |  | If “No” *Mark and Go to Q 7.2* | If “Unknown” Mark and Go to Q 7.2 |
| **2** | **Do you feed any dogs that you do not own? (7.2)**  ***If “Yes”* : how many dogs? (7.2.1) _____**  ***If “Yes”:* Do you know if someone owns the dogs? (7.2.2) (choose one)**   - 1. **Yes, the dogs are owned by someone.**   2. **No, the dogs are stray dogs**   3. **Do not know** |  | If “No” Mark and Go to Q 7.3 | If “Unknown” Mark and Go to Q 7.3 |
| **3** | **Do you have any Cats (7.3)**  ***If “Yes”: How many cats? (7.3.1)***  ***If “Yes”:* Are they kept in the house all the time? (7.3.2)**  **1.Yes**  **2.No** |  | If “No” Mark and Go to Q 7.4 | If “Unknown” Mark and Go to Q 7.4 |
| **4** | **Do you have any Cattle (cow, buffalo ...) (7.4)** |  |  |  |
| **5** | **Do you have any Small ruminants (sheep, goat ...) (7.5)** |  |  |  |
| **6** | **Do you have any solid-hoofed animals (horse, donkey, mule) (7.6)** |  |  |  |
| **7** | **Do you have any Pigs (7.7)** |  |  |  |
| **8** | **Do you have any Wild animals (7.8)** |  |  |  |
| **9** | **Do you have any Other animals? (7.9) Specify** | | | |

1. **Do you know what responsibility do you assume according to the legislation when you are keeping cats and dogs? (8.0)**
2. Yes
3. No
4. Partially
5. **Have you noticed stray dogs in your city/village? (9.0)**
6. Yes
7. No (Go to Q11)
8. Unknown (Go to Q11)
9. **If “yes”, how many? (10.0)**
10. 1-3
11. 4-5
12. More than 5
13. **Have you noticed stray cats in your city/village? (11.0)**
14. Yes
15. No (Go to Q13)
16. Unknown (Go to Q13)
17. **If “yes”, how many? (12.0)**
18. 1-3
19. 4-5
20. More than 5
21. **Have attacks of wild animals to humans or to domestic animals been reported in your town/village? (13.0)**
22. Yes
23. No (Go to Q 15)
24. I don’t know (Go to Q15)
25. **If, yes, who was a target of this attack? (14.0)**
26. Human
27. Animal
28. **Have you personally had contact (bite, scratch, lick) from wild animals? (15.0)**
29. Yes
30. No (Go to Q 18)
31. **With which kind of wild animal did you have contact (bite, scratch, lick)? (16.0)**

|  |  | **Yes** | **No** | **Unknown** |
| --- | --- | --- | --- | --- |
| **1** | **Wolf (16.1)** |  |  |  |
| **2** | **Bear (16.2)** |  |  |  |
| **3** | **Fox (16.3)** |  |  |  |
| **4** | **Jackal (16.4)** |  |  |  |
| **5** | **Bat (16.5)** |  |  |  |
| **6** | **Other (SPECIFY) (16.6)** | | | |

1. **In which circumstances did you have contact with a wild animal (17.0) (Circle all named )**
2. During hunting (17.1) (Y)
3. While walking around in a city / village (17.2) (Y)
4. While at my private agricultural plot (17.3) (Y)
5. When an attack was made on my animals (17.4) (Y)

**Rabies Knowledge**

1. **Do you know what rabies is? (18.0)**
2. Yes
3. No
4. **From where do get information on rabies or other diseases? (19.0)**

|  |  | **Yes** | **No** | **Unknown** |
| --- | --- | --- | --- | --- |
| **1** | **From private veterinarian (19.1)** |  |  |  |
| **2** | **From government veterinarian (19.2)** |  |  |  |
| **3** | **From my friend/relative (19.3)** |  |  |  |
| **4** | **Written Press (newspapers, magazines) (19.4)** |  |  |  |
| **5** | **TV (19.5)** |  |  |  |
| **6** | **Radio (19.6)** |  |  |  |
| **7** | **Written pamphlets (19.7)** |  |  |  |
| **8** | **Posters in community buildings (19.8)** |  |  |  |
| **9** | **Text messages on my phone (19.9)** |  |  |  |
| **10** | **Social networks (facebook, twitter) (19.10)** |  |  |  |
| **11** | **Other internet websites (19.11)** |  |  |  |
| **12** | **Other sources of information (19.12) (SPECIFY)** | | | |

1. **Do you know what are the symptoms of animal rabies? (20.0)**
2. Yes
3. No (Go to Q 22)
4. **If ,,yes’’, please specify (DO NOT read, circle ) (21.0)**
5. Change in behavior (21.1) (Y)
6. Loss of appetite (21.2) (Y)
7. Fever (21.3) (Y)
8. Disorientation (21.4) (Y)
9. Salivation (21.5) (Y)
10. Unprovoked aggression (21.6) (Y)
11. Paralysis (21.7) (Y)
12. Weakness (21.8) (Y)
13. Choking livestock (21.9) (Y)
14. Unconsciousness (21.10)(Y)
15. Other. (21.11) (SPECIFY) ________________________________________
16. **Have any of your animals, or animals of your friends/relatives died with any of the above-mentioned symptoms (change in behavior, loss of appetite, fever, disorientation, aggression, gnawing of subjects) within the last year? (22.0)**
17. Yes
18. No
19. I don’t know

**23. Have you notifed a private or a government veterinarian in case of observation of any of the above-mentioned symptoms? (Choose one) (23.0)**

1. I have not observed any
2. I have observed but I have not notified anyone
3. I have observed, and I have notified a private veterinarian
4. I have observed and I have notified a government veterinarian
5. I have observed and I have notified both a private and a government veterinarian.

**24. Have you notifed anyone else in case of observation of any of the above-mentioned symptoms?** (24.0)

|  |  | **Yes** | **No** | **Unknown** |
| --- | --- | --- | --- | --- |
| **1** | **Doctor** (24.1) |  |  |  |
| **2** | **Family member** (24.2) |  |  |  |
| **3** | **Police Officer** (24.3) |  |  |  |
| **4** | **Teacher** (24.4) |  |  |  |
| **5** | **Other (SPECIFY)** (24.5) | | | |

**25. What should be done in order to protect a domestic animal from rabies? (25.0) (Choose one)**

1. One time lifelong vaccination
2. Regular vaccination, according to the prescription of the vet
3. There are no means of animal protection from rabies
4. I do not know
5. Other (Specify) ________________

**26. In your opinion, which animal is the source of spread of animal rabies? (26.0)**

|  |  | **Yes** | **No** | **Unknown** |
| --- | --- | --- | --- | --- |
| **1** | **Pet dogs (26,1)** |  |  |  |
| **2** | **Pet cats (26.2)** |  |  |  |
| **3** | **Wild animals (wolves, foxes, jackals ...) (26.3)** |  |  |  |
| **4** | **Stray dogs (26.4)** |  |  |  |
| **5** | **Stray cats (26.5)** |  |  |  |

**Rabies Vaccination**

**27. Has the anti-rabies vaccination campaign for animals ever been conducted in your village/town? (27.0)**

1. Yes
2. No
3. I don’t know

**28.Have you vaccinated your livestock against rabies? (28.0)**

1. Yes
2. No (Go to Q 31)
3. I don’t know/I don’t remember (Go to Q 31)
4. Not applicable (N/A: do not have livestock)

**29. If “Yes” how? (29.0)**

|  |  | **Yes** | **No** | **Unknown** |
| --- | --- | --- | --- | --- |
| **1** | **On my/family member’s own initiative (29.1)** |  |  |  |
| **2** | **During anti-rabies vaccination campaign (29.2)** |  |  |  |
| **3** | **During “ring vaccination” after the case was registered in the village (29.3)** |  |  |  |
| **4** | **Private vets regular visit (29.4)** |  |  |  |
| **5** | **Governmental vets regular visit (29.5)** |  |  |  |

**30. If ,,yes’’, how often? (30.0)**

1. Annually
2. Once
3. I do not remember

**31. Have you vaccinated your dog against rabies? (31.0)**

1. Yes
2. No (Go to Q 34)
3. I don’t know/I don’t remember (Go to Q 34)
4. Not applicable (N/A: do not have a dog)

**32. If “Yes” how? (32.0)**

|  |  | **Yes** | **No** | **Unknown** |
| --- | --- | --- | --- | --- |
| **1** | **On my/family member’s own initiative (32.1)** |  |  |  |
| **2** | **During anti-rabies vaccination campaign (32.2)** |  |  |  |
| **3** | **During “ring vaccination” after the case was registered in the village (32.3)** |  |  |  |
| **4** | **Private vets regular visit (32.4)** |  |  |  |
| **5** | **Governmental vets regular visit (32.5)** |  |  |  |

**33. If ,,yes’’, how often? (33.0)**

1. Annually
2. Once
3. I do not remember

**34. If you have not vaccinated your dog, please state the reasons (34.0) (Choose one)**

1. I do not know how mandatory is to vaccinate an animal against rabies
2. Because of the high cost of the vaccine
3. The vet is not located close enough
4. I was not able to catch the dog to permit vaccination
5. I don’t think it is necessary
6. I don’t know

**35. Have you vaccinated your cat against rabies? (35.0)**

1. Yes
2. No (Go to Q 38)
3. I don’t know/I don’t remember (Go to Q 38)
4. Not applicable (N/A: do not have a cat)

**36. If “Yes” how? (36.0)**

|  |  | **Yes** | **No** | **Unknown** |
| --- | --- | --- | --- | --- |
| **1** | **On my/family member’s own initiative (36.1)** |  |  |  |
| **2** | **During anti-rabies vaccination campaign (36.2)** |  |  |  |
| **3** | **During “ring vaccination” after the case was registered in the village (36.3)** |  |  |  |
| **4** | **Private vets regular visit (36.4)** |  |  |  |
| **5** | **Governmental vets regular visit (36.5)** |  |  |  |

**37. If ,,yes’’, how often? (37.0)**

1. Annually
2. Once
3. I do not remember

**38. If you have not vaccinated your cat, please state the reasons (38.0) (Choose one)**

1. I do not know how mandatory is to vaccinate an animal against rabies
2. Because of the high cost of the vaccine
3. The vet is not located close enough
4. I was not able to catch the cat to permit vaccination
5. I don’t think it is necessary
6. I don’t know

**39. Who has vaccinated your dog/cat? (39.0)**

1. Veterinarian
2. Myself
3. Other persons

**40. Do you have an animal vaccination confirming document (40.0)**

1. Yes
2. No
3. I don’t know

**41. Have any animal rabies cases been reported in your town/village within the last year? (41.0)**

1. Yes
2. No (Go to Q 43)
3. Don’t know (Go to Q 43)

**42. If ,,yes’’, was anti-rabies vaccination conducted among animals for this specific case? (42.0)**

1. Yes
2. No
3. I don’t know

**43. Has you animal ever bitten/scratched/ licked to any human? (43.0)**

1. Yes
2. No (Go to Q 45)
3. C.I do not have animals (Go to Q45)
4. I don’t know (Go to Q 45)

**44. If ,,yes’’, has a vet provided 10-day supervision over your animal? (44.0)**

1. Yes
2. No
3. I don’t know
4. I was supervising myself; if necessary I would have notified a vet.

**45. In your opinion, what methods/measures should be taken to prevent spread of rabies among animals? (45.0)**

|  |  | **Yes** | **No** | **Unknown** |
| --- | --- | --- | --- | --- |
| **1** | **Anti-rabies mass vaccination of domestic carnivores (45.1)** |  |  |  |
| **2** | **Control of stray animals through sterilization (45.2)** |  |  |  |
| **3** | **Euthanasia of stray animals (45.3)** |  |  |  |
| **4** | **Anti-rabies mass vaccination of stray animals (45.4)** |  |  |  |
| **5** | **Anti-rabies vaccination of wild animals (45.5)** |  |  |  |
| **6** | **Raising awareness of population about rabies (45.6)** |  |  |  |
| **7** | **I don’t know (45.7)** |  |  |  |
| **8** | **Other. (45.8) (SPECIFY)** |  | | |

**Human Practices**

**46. In your opinion, how does a human get infected with rabies (46.0)**

|  |  | **Yes** | **No** | **Unknown** |
| --- | --- | --- | --- | --- |
| **1** | **From saliva of sick animals onto damaged human skin (46.1)** |  |  |  |
| **2** | **From bite of sick animal (46.2)** |  |  |  |
| **3** | **From scratch of a sick animal (46.3)** |  |  |  |
| **4** | **Through breathing (Airborne route) near a sick animal (46.5)** |  |  |  |
| **5** | **Feces or blood of sick animals (46.6)** |  |  |  |
| **6** | **Through other physical contact with sick animals (46.4) (SPECIFY)** | | | |

**47. Disease develops faster if a bite occurred in which part of the body (47.0) (Choose one)**

1. The upper extremities (arms)
2. The lower extremities (legs)
3. Trunk (chest/back)
4. Head
5. Don’t know

**48. What are the symptoms of human rabies? (DO NOT read) (48.0) (circle all named)**

1. Weakness, discomfort, fever, headache (48.1) (Y )
2. Itching at a bite region (48.2) ( Y )
3. Neural signs (48.3) ( Y )
4. Anxiety (48.4) ( Y )
5. Confusion (48.5) ( Y )
6. Delirium (48.6) ( Y )
7. Abnormal Behavior (48.7) ( Y)
8. Hallucinations (48.8) ( Y )
9. Insomnia (48.9) ( Y )
10. Hydrophobia (48.10) ( Y )
11. Other (48.11) (SPECIFY) _____________________________

**49. Is it possible to prevent human rabies? (49.0)**

1. Yes
2. No (Go to Q 51)
3. don’t know (Go to Q 51)

**50. If ,,ye s’’, how (Do not read) (50.0) (Circle all named)**

1. Vaccination before biting (routine vaccination) (50,1) (Y)
2. Post-biting vaccination (post-exposure vaccination) (50,2) (Y)
3. Just treatment of bite region (50,3) (Y)
4. Folk remedies (50,4) (Y)
5. I do not know (50,5) (Y)
6. Other (50,6) (SPECIFY) _________________________________

**51. Is rabies treatable among humans after manifestation of clinical signs (51.0)**

1. Yes
2. No (Go to Q 53)
3. Don’t know (Go to Q 53)

**52. If ,,yes’’, how is rabies treatable? (52.0)**

|  |  | **Yes** | **No** | **Unknown** |
| --- | --- | --- | --- | --- |
| **1** | **Vaccination (52.1)** |  |  |  |
| **2** | **Drug therapy (52.2)** |  |  |  |
| **3** | **Folk remedies (52.3)** |  |  |  |
| **4** | **I do not know (52.4)** |  |  |  |
| **5** | **Other(SPECIFY) (52.5)** | | | |

**53.In which period from exposure should immunization start to avoid the disease? (53.0) (Choose one)**

1. As soon as possible within the first two days after contact with animals (biting, saliva, scratching)
2. Within 3-14 days
3. From 15 days onward
4. Any time
5. I do not know

**54. Have you ever been bitten/ scratched/ licked of an animal? (54.0)**

1. Yes
2. No (Go to Q 57)
3. Don’t know (Go To Q 57)

**55. IF YES what kind of animal was it?(55.0)**

|  |  | **Yes** | **No** |
| --- | --- | --- | --- |
| **1** | **your own dog (55.1)** |  |  |
| **2** | **your own cat (55.2)** |  |  |
| **3** | **a stray dog (55.3)** |  |  |
| **4** | **a stray cat (55.4)** |  |  |
| **5** | **Livestock (55.5)** |  |  |
| **6** | **Someone else's dog (55.6)** |  |  |
| **7** | **a wild animal (55.7)** |  |  |
| **8** | **Other (SPECIFY) (55.8)** | | |

**56. If you were bitten, scratched, or exposed to saliva’, did you visit a doctor? (56.0)**

1. Yes
2. No (Go to Q 59)
3. I don’t remember.

**57. Have you been vaccinated against rabies? (57.0)**

1. Yes
2. No (Go to Q 59)
3. I don’t know (Go to Q 59)

**58.Do you have a vaccination confirming document (58.0)**

1. Yes
2. No
3. I don’t know

**59.Will you visit a doctor in case of a possible future bite/scratch/licked of an animal (59.0)**

1. Yes
2. No
3. I don’t know

**60. If a family member was bitten, scratched, or licked by an animal, would you take them to a doctor? (60.0)**

1. Yes
2. No
3. I don’t know

**61. If “no” - why?(61.0)**

|  |  | **Yes** | **No** |
| --- | --- | --- | --- |
| **1** | **Lack of funds (60,1)** |  |  |
| **2** | **Medical center is located a great distance away (60,2)** |  |  |
| **3** | **My family member is a doctor and he/she will take care of me (60,3)** |  |  |
| **4** | **I do not think that it is necessary/I'm not afraid/I'm strong enough to survive (60.4)** |  |  |
| **5** | **I can not go to the doctor for all biting episodes (60,5)** |  |  |
| **6** | **I do not think that it is necessary; I have gone through the vaccination during the previous biting episode (60,6)** |  |  |
| **7** | **Animal that bites is my own animal and it is vaccinated against rabies (60,7)** |  |  |
| **8** | **Other (60,8) (SPECIFY)** | | |

**Thank you for information!**
